# Higher content of microcystin‐leucine‐arginine promotes the survival of intrahepatic cholangiocarcinoma cells via regulating SET resulting in the poorer prognosis of patients

**DOI:** 10.1111/cpr.12961

**Published:** 2020-11-25

**Authors:** Shen Gu, Wei He, Minghao Yan, Jian He, Qun Zhou, Xiaopeng Yan, Xiao Fu, Jun Chen, Xiaodong Han, Yudong Qiu

**Affiliations:** ^1^ Department of Hepatopancreatobiliary Surgery Drum Tower Hospital Medical School of Nanjing University Nanjing China; ^2^ Immunology and Reproduction Biology Laboratory & State Key Laboratory of Analytical Chemistry for Life Science Medical School Nanjing University Nanjing China; ^3^ Jiangsu Key Laboratory of Molecular Medicine Nanjing University Nanjing China; ^4^ Department of Clinical Pharmacology Key Laboratory of Clinical Cancer Pharmacology and Toxicology Research of Zhejiang Province Hangzhou First People's Hospital Hangzhou China; ^5^ Department of Neurosurgery Affiliated Aoyang Hospital of Jiangsu University Zhangjiagang China; ^6^ Department of Radiology Nanjing Drum Tower Hospital The Affiliated Hospital of Nanjing University Medical School Nanjing China; ^7^ Department of Pathology Nanjing Drum Tower Hospital The Affiliated Hospital of Nanjing University Medical School Nanjing China

**Keywords:** intrahepatic cholangiocarcinoma, microcystin‐leucine‐arginine, PP2A, prognosis, SET

## Abstract

**Background & Aims:**

Intrahepatic cholangiocarcinoma (ICC) has over the last 10 years become the focus of increasing concern largely due to its rising incidence and high mortality rates worldwide. Microcystin‐leucine‐arginine (MC‐LR) has been reported to be carcinogenic, but there are no data on the linkage between MC‐LR and ICC. This study aimed to explore whether the content levels of MC‐LR in the tumour tissues of ICC patients be associated with the prognosis and if so, to characterize the mechanism in ICC cells.

**Methods:**

We conducted a retrospective study to evaluate the prognostic value of MC‐LR in ICC after resection. All patients were divided into two groups according to the content of MC‐LR in tumour via immunohistochemistry: low‐MC‐LR group (n = 28) and high‐MC‐LR group (n = 30).

**Results:**

Multivariate analysis showed high‐MC‐LR level was the prognostic factor for OS and RFS after hepatectomy (*P* = .011 and .044). We demonstrated that MC‐LR could promote the survival of human ICC cell lines and SET was identified as an important mRNA in the progression via RNA array.

**Conclusions:**

We provide evidence that MC‐LR was an independent prognostic factor for ICC in humans by modulating the expression of SET in human ICC cells.

## Graphical abstract

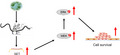

## 1 INTRODUCTION

Intrahepatic cholangiocarcinoma (ICC) accounts for 5%‐10% of primary liver cancers.^1^ Compared to Western countries, the incidence rate of ICC in Eastern Asia was much higher.^2^ The recognizable risk factors for ICC have yet been entirely identified, resulting in that ICCs are frequently diagnosed at an advanced stage.^3^ Although the management options and standard of care for ICC have gained great advances in the last decades, the prognosis for ICC remains dismal.^4^ Compared to hepatocellular carcinoma (HCC), mass‐forming ICCs are typically characterized by an extreme tumour stroma.^5^

Microcystins (MCs) are a series of environmental hepatotoxins produced by cyanobacteria. MCs have been confirmed to be possible tumour promoters and carcinogens in vivo and vitro.^6^ Because of its strong inhibition of protein phosphatase 1 and 2A (PP1 and PP2A), microcystin‐leucine‐arginine (MC‐LR), one of the most widely studied and poisonous MCs in water bodies, is considered to be tumour promoter in mammals.^7^, ^8^ Protein Phosphatase 2A (PP2A) is an important and ubiquitously expressed serine threonine phosphatase and regulates the function by dephosphorylating many critical cellular molecules. It plays a critical role in cellular processes, such as cell proliferation, signal transduction and apoptosis, highlighting its role as a ‘tumour suppressor’. Previous studies have confirmed that populations exposed to MCs through their drinking water own a higher liver carcinoma mortality rate than populations who drink well water.^9^ Serum MC‐LR level was also confirmed as an independent risk factor for HCC in humans.^10^ However, the influence of exposure to MCs through food and drinking water on the progression of ICC is relatively less studied.

SET is also known as a specific inhibitor of PP2A.^11^ Overexpression of SET has been demonstrated in head and neck squamous cell carcinoma (HNSCC) and promotes HNSCC cell survival, proliferation and resistance to cell death by cisplatin in vivo.^12^, ^13^ The phosphorylation of extracellular signal‐regulated kinase (ERK) and protein kinase B (Akt) was frequently observed in the SET‐mediated PP2A inhibition process.^14^, ^15^ It is reported that MEK/ERK signalling pathway participates in adrenocortical tumorigenesis that could be potentially used as a diagnostic marker for malignancy.^16^ Increasing the level of phosphorylated‐MEK could result in the activation of ERK1/2 via phosphorylation and translocation to the nucleus.^17^

Our main objective was to analyse the connection between the content level of MC‐LR in the tumour tissues of ICC patients and other clinical data. We also endeavoured to identify the contribution of MC‐LR to the progression of ICC in vivo and investigate the mechanism in this procedure.

## 2 MATERIALS AND METHODS

### 2.1 Main materials

MC‐LR was acquired from Alexis Biochemicals.

### 2.2 Patients and tumour samples

From February 2005 to March 2016, a total of 138 consecutive intrahepatic cholangiocarcinoma (ICC) patients experienced anatomic liver resection in our institution. To accurately evaluate the prognostic impact of MC‐LR in ICC, the following exclusion criteria were presented: (a) lack of tissue slice, (b) R1 tumour resection, (c) incomplete prognostic or clinical data and (d) a history of other cancers (Figure S1). Accordingly, a total of 58 patients were included in the prognosis analysis. In the meanwhile, to clearly predict the content levels of MC‐LR through image data, the inclusion criteria were as follows: (a) with preoperative contrast‐enhanced computed tomography (CT) imaging in our hospital, (b) with single lesion and (c) without any systematic or local treatment before CT examination. Finally, 43 patients were included for image data analysis. The present study was carried out in accordance with the Declaration of Helsinki revised in 1983 and approved from the requirement to obtain informed consent by the Committee on Medical Ethics of Nanjing Drum Tower Hospital. The course for patients was better described in the Appendix S1.

### 2.3 Tissue microarray blocks (TMAs)

The tissue microarray was used for detection of MC‐LR according to our previous study.^18^ We have also provided a detailed description in the Appendix S1.

### 2.4 Imaging techniques

All patients underwent CT examination as previously described.^18^ The detailed description has been provided in the Appendix S1.

### 2.5 Imaging analysis

The imaging analysis was conducted as the procedure in our previous study.^18^ The detailed description has been provided in the Appendix S1.

### 2.6 Immunohistochemical analyses

The experiment was performed as previously described.^18^ The following primary antibodies were employed: rabbit anti‐SET (Cell Signaling Technology) and mouse anti‐MC‐LR (Enzo). We have also provided a detailed protocol in the Appendix S1.

### 2.7 Cell culture

Immortalized human ICC cell lines (huh28 and RBE) were used according to the previous reports.^19^, ^20^ The detailed description has been supported in the Appendix S1.

### 2.8 Flow cytometric analysis

The apoptosis assay was detected according to the manufacturer's instructions. The detailed description has been provided in the Appendix S1.

### 2.9 Proliferation assay

Cell proliferation was analysed by 5‐ethynyl‐2′‐deoxyuridine (EdU) incorporation using the Click‐iT EdU Alexa Fluor 488 cell proliferation assay kit (Invitrogen). We have also provided a detailed protocol in the Appendix S1.

### 2.10 Cell migration assays

Cell migration assay was used to determine the ability of cell metastasis. For the migration assay, 1 × 10^5^ cells were added into the upper chamber of an 8‐μm pore sized polycarbonate nucleopore filter inserts in a 24‐well Transwell chamber (Corning Costar). The detailed description has been provided in the Appendix S1.

### 2.11 Immunofluorescent staining

Immunofluorescence analysis was performed as described previously.^21^ The following primary antibodies were employed: mouse anti‐MC‐LR (Enzo). Alexa Fluor 488‐conjugated goat anti‐rabbit (Invitrogen) was used as the secondary antibody. The images were captured using a confocal fluorescence microscope (Olympus).

### 2.12 RNA purification

Cells were lysed via the TRIzol reagent (Invitrogen), and total RNA was isolated for mRNA evaluation.

### 2.13 Profiling RNA with Affymetrix GeneChip^®^ Human Genome U133 Plus 2.0 Array

Cells incubated in the absence or presence of 500 nmol/L MC‐LR for 24 hours were harvested, and total RNA was isolated immediately from 10^6^ cells. The mRNA microarray analysis was performed by Affymetrix GeneChip^®^ Human Genome U133 Plus 2.0 Array according to the manufacturer's instructions. Data were analysed as previously described.^22^ mRNAs with expression values of less than 0.5 (log scale)‐fold change compared to controls were considered as under‐expressed, while those with a value of greater than 2 were regarded as overexpressed. Each group contained three technical replicates.

### 2.14 Quantitative real‐time polymerase chain reaction (Q‐PCR)

The sequences of primer pairs used in this assay are shown in Table S4, and total RNA was isolated from cells in three independent culture plates and analysed in triplicate using Q‐PCR. The detailed description has been provided in the Appendix S1.

### 2.15 Selected small interfering RNA and transfection condition

The preliminary studies of the project showed that the siRNA targeting to SET (target sequence: GGTGATCCATCTTCGAAGT, sense: 5′‐GGUGAUCCAUCUUCGAAGUdTdT‐3′ and antisense: 3′‐dTdTCCACUAGGUAGAAGCUUCA‐5′) achieved the highest knockdown effect among three designed siRNAs and was selected for the following experiments (Figure S2). The detailed description has been provided in the Appendix S1.

### 2.16 Western blotting

Proteins were harvested from huh28 and RBE cells. Western blotting analysis was performed as previously described.^23^ The following primary antibodies were employed: Rabbit anti‐ITK, rabbit anti‐SET, mouse anti‐GAPDH, rabbit anti‐ERK1/2, rabbit anti‐p‐ERK1/2, rabbit anti‐Akt, rabbit anti‐p‐Akt, rabbit anti‐MEK1/2, rabbit anti‐p‐MEK1/2 (Cell Signaling Technology) and mouse anti‐MC‐LR (Enzo). We have also provided a particular procedure in the Appendix S1.

### 2.17 Luciferase reporter assay

Luciferase activity assay was performed using the Dual‐Luciferase Reporter Assay System (Promega) according to the manufacturer's instructions. ICC cells were transfected with the plasmids containing the pLG3 vector (Obio Technology) inserted with promotor sequence of SET (NM_001122821). The cells were treated with MC‐LR at 500 nmol/L 24 hours after transfection, and the luciferase activity assay was performed 24 hours after exposure to MC‐LR via the Dual‐Luciferase Assay System.

### 2.18 PP2A activity assay

PP2A activity assay was performed by using a PP2A immunoprecipitation phosphatase assay (Millipore) according to the manufacturer's instructions. Total protein from ICC cells, transiently transfected with siRNA‐NC and siRNA‐SET followed by exposure in the presence or absence of MC‐LR, was extracted with lysis buffer. We have also provided a particular procedure in the Appendix S1.

### 2.19 Statistical analysis

For all tests, *P* values < .05 were considered statistically significant. Statistical analysis was performed using SPSS version 22.0 (SPSS Inc). The detailed description has been provided in the Appendix S1.

## 3 RESULTS

### 3.1 Comparison of patient characteristics and prognosis according to the content level of MC‐LR

Based on the results of tissue microarray blocks (TMAs), we defined three content levels of MC‐LR: level 0 (n = 6), level 1 (n = 22) and level 2 (n = 30) (Figure S3). All ICC patients were divided into two groups according to the content level of MC‐LR: low‐MC‐LR (n = 28), containing patients defined as level 0 and level 1, and high‐MC‐LR (n = 30), containing patients defined as level 2. Clinical and pathological characteristics of the two groups were summarized in Table S1. Gender and mass‐forming type rate in low‐MC‐LR group were significantly different compared with high‐MC‐LR groups (*P* = .031 and *P* = .049), while there were no significant differences in other characteristics. The overall survival (OS) rate in high‐MC‐LR group was obviously lower than that in low‐MC‐LR group via Kaplan‐Meier analyses (*P* = .009). Similarly, the recurrence‐free survival (RFS) rate in high‐MC‐LR group was significantly poorer than that in low‐MC‐LR group (*P* = .031) (Figure S4).

### 3.2 Univariate and multivariate analyses of survival and recurrence in ICC patients after hepatectomy

Univariate and multivariate analyses of predictors for OS and RFS rates in ICC patients were illustrated in Tables 1, 2. Univariate analysis indicated that spread along the Glisson pedicles, tumour staging and MC‐LR was the risk factors for OS rate. Multivariate analysis identified tumour staging (HR, 0.151 [95% CI, 0.042‐0.551], *P* = .004) and MC‐LR (HR, 0.310 [95% CI, 0.126‐0.766], *P* = .011) as independent prognostic factors. Additionally, the RFS rate was affected by tumour staging and MC‐LR in the univariate analysis. Multivariate analysis identified tumour staging (HR, 0.389 [95% CI, 0.181‐0.834], *P* = .015), MC‐LR (HR, 0.489 [95% CI, 0.244‐0.982], *P* = .044) as independent prognostic factors.

**TABLE 1:** Univariate and multivariate analysis of risk factors for overall survival rate

Variable	Univariate analysis		Multivariate analysis	
	HR (95% CI)	*P*‐value	HR (95% CI)	*P*‐value
Age (≤60 y vs >60 y)	0.563 (0.248‐1.280)	.170		
Gender (male vs female)	0.661 (0.291‐1.503)	.324		
SGS (yes vs no)	0.313 (0.132‐0.740)	.008	0.908 (0.290‐2.845)	.868
Tumour number (single vs multi)	0.783 (0.308‐1.990)	.607		
Tumour staging (I & II vs III & IV)	0.159 (0.059‐0.430)	<.001	0.151 (0.042‐0.551)	.004
Tumour size (≤5 cm vs >5 cm)	1.809 (0.780‐4.197)	.167		
ALT (normal or abnormal)	0.769 (0.316‐1.871)	.563		
AST (normal or abnormal)	0.781 (0.320‐1.904)	.586		
AKP (normal or abnormal)	0.897 (0.304‐2.645)	.844		
GGT (normal or abnormal)	0.412 (0.160‐1.056)	.065		
LDH (normal or abnormal)	1.019 (0.302‐3.440)	.976		
TB (normal or abnormal)	0.995 (0.368‐2.692)	.993		
DB (normal or abnormal)	0.645 (0.190‐2.188)	.481		
Albumin (normal or abnormal)	0.660 (0.289‐1.508)	.324		
AFP (normal or abnormal)	0.630 (0.186‐2.134)	.458		
CA19‐9 (normal or abnormal)	0.470 (0.198‐1.115)	.087		
CEA (normal or abnormal)	0.773 (0.177‐3.373)	.732		
MC‐LR (low content vs high content)	0.336 (0.141‐0.800)	.014	0.310 (0.126‐0.766)	.011

Abbreviations: AFP, alpha‐fetoprotein; AKP, alkaline phosphatase; ALT, alanine aminotransferase; AST, Aspartate aminotransferase; CA19‐9, carbohydrate antigen 19‐9; CEA, carcinoembryonic antigen; DB, direct bilirubin; GGT, gamma glutamyl transpeptidase; LDH, lactate dehydrogenase; MC‐LR, microcystins‐leucine‐arginine; SGS, spread along the Glisson pedicles; TB, total bilirubin.

**TABLE 2:** Univariate and multivariate analysis of risk factors for recurrence‐free survival rate

Variable	Univariate analysis		Multivariate analysis	
	HR (95% CI)	*P*‐value	HR (95% CI)	*P*‐value
Age (≤60 y vs >60 y)	0.606 (0.312‐1.180)	.141		
Gender (male vs female)	0.988 (0.507‐1.924)	.972		
SGS (yes vs no)	0.517 (0.259‐1.032)	.062		
Tumour number (single vs multi)	0.715 (0.324‐1.578)	.407		
Tumour staging (I & II vs III & IV)	0.383 (0.181‐0.814)	.013	0.389 (0.181‐0.834)	.015
Tumour size (≤5 cm vs >5 cm)	1.350 (0.694‐2.627)	.377		
ALT (normal or abnormal)	0.850 (0.397‐1.871)	.674		
AST (normal or abnormal)	0.987 (0.448‐2.175)	.974		
AKP (normal or abnormal)	1.423 (0.502‐4.035)	.507		
GGT (normal or abnormal)	0.569 (0.278‐1.163)	.122		
LDH (normal or abnormal)	0.862 (0.304‐2.445)	.780		
TB (normal or abnormal)	0.995 (0.368‐2.692)	.993		
DB (normal or abnormal)	1.243 (0.380‐4.063)	.719		
Albumin (normal or abnormal)	1.298 (0.660‐2.554)	.450		
AFP (normal or abnormal)	0.671 (0.236‐1.906)	.453		
CA19‐9 (normal or abnormal)	0.576 (0.288‐1.153)	.119		
CEA (normal or abnormal)	1.257 (0.383‐4.129)	.706		
MC‐LR (low content vs high content)	0.483 (0.243‐0.961)	.038	0.489 (0.244‐0.982)	.044

Abbreviations: AFP, alpha‐fetoprotein; AKP, alkaline phosphatase; ALT, alanine aminotransferase; AST, Aspartate aminotransferase; CA19‐9, carbohydrate antigen 19‐9; CEA, carcinoembryonic antigen; DB, direct bilirubin; GGT, gamma glutamyl transpeptidase; LDH, lactate dehydrogenase; MC‐LR, microcystins‐leucine‐arginine; SGS, spread along the Glisson pedicles; TB, total bilirubin.

### 3.3 Predictive value of preoperative imaging factors for the content level of MC‐LR

We investigated the computed tomography (CT) values in equilibrium phase of lesions for predicting the content level of MC‐LR (Table 3). The significant predictors (*P* < .1) in univariate analysis were entered into the multivariate logistic regression model to identify the valuable independent predictors. The ratio between the max and mean tumour CT values in equilibrium phases (tMax/tMean) was independent predictor for content level of MC‐LR in tumour (OR = 0.017, 95% CI 0.001‐0.546, *P* = .021). Based on ROC curve analysis of the prediction score, the area under receiver operating characteristic (AUROC) was 0.710 (95% CI, 0.548‐0.871). The optimal cut‐off score was 1.38 on the basis of maximum Youden index value (Figure S5).

**TABLE 3:** Predictors for high‐MC‐LR content level of cholangiocarcinoma on univariate analysis

Variable	Low‐MC‐LR (n = 28)	High‐MC‐LR (n = 15)	OR (95% CI)	*P*‐value
Age				
≤60	12 (42.9)	5 (33.3)	0.667 (0.180‐2.468)	.544
>60	16 (57.1)	10 (66.7)		
Gender				
Male	15 (53.6)	8 (53.3)	0.990 (0.282‐3.482)	.988
Female	13 (46.4)	7 (46.7)		
Gross Classification				
MF	20 (71.4)	10 (66.7)	0.800 (0.207‐3.088)	.746
Non‐MF	8 (28.6)	5 (33.3)		
Enhanced ratio				
≤2/3	16 (57.1)	14 (93.3)	10.500 (1.208‐91.268)	.033
>2/3	12 (42.9)	1 (6.7)		
tMean				
≤77	4 (14.3)	9 (60)	9.000 (2.051‐39.498)	.004
>77	24 (85.7)	6 (40)		
tMax				
≤116	11 (39.3)	12 (80)	6.182 (1.414‐27.023)	.016
>116	17 (60.7)	3 (20)		
tMax – tMean				
≤25	11 (39.3)	3 (20)	0.386 (0.088‐1.689)	.206
>25	17 (60.7)	12 (80)		
tMax/tMean				
≤1.38	16 (57.1)	3 (20)	0.188 (0.043‐0.815)	.026
>1.38	12 (42.9)	12 (80)		

Abbreviations: MC‐LR, microcystins‐leucine‐arginine; tMax, max CT value of tumour in equilibrium phases; tMean, mean CT value of tumour in equilibrium phases.

### 3.4 Influence of MC‐LR on human ICC cell lines

**FIGURE 1:**
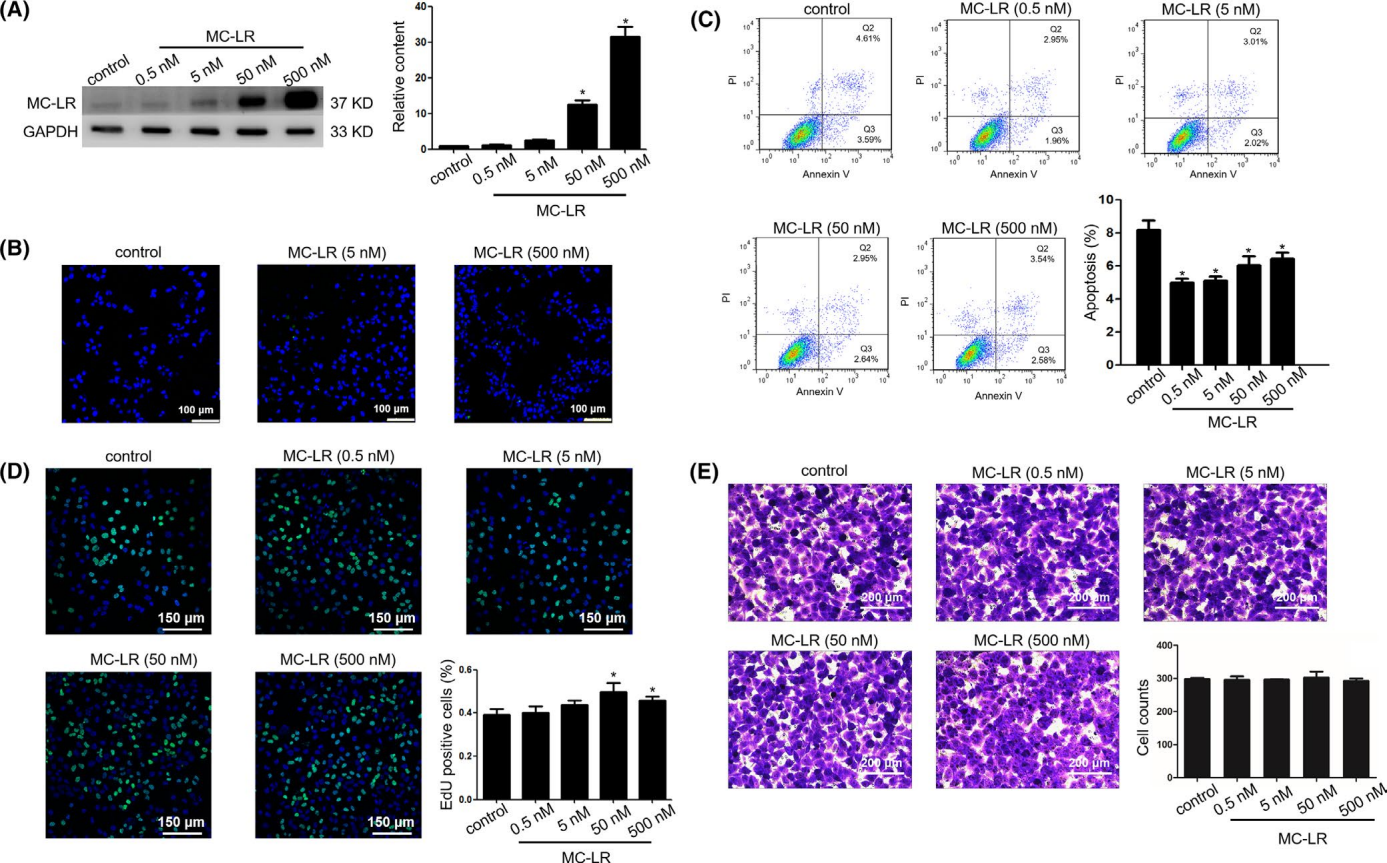
Microcystin‐leucine‐arginine (MC‐LR) can be uptaken into huh28 cells inducing cell survival of intrahepatic cholangiocarcinoma cells. Huh28 cells were cultured in the presence or the absence of MC‐LR at indicated concentrations for 24 h. A, The content level of MC‐LR in huh28 cells was determined by Western Blotting. B, Intracellular MC‐LR was detected by immunofluorescent confocal microscopy (200×). Green colour indicates the expression of MC‐LR. C, Cell apoptosis analysis in huh28 cells was conducted through flow cytometry (lower panel). D, Measurement of cell viability was carried out with the 5‐ethynyl‐2′‐deoxyuridine (EdU) incorporation assay. E, The migration of the huh28 cells treated with MC‐LR was measured through crystal violet's staining. MC‐LR was quantified by densitometry and normalized to the expression of GAPDH. Three repeats at least were performed in all experiments for three times. *P < .05 vs control

Huh28 cells were exposed to MC‐LR at various concentrations for 24 hours followed by measurement of MC‐LR via Western Blotting and immunohistochemical staining. MC‐LR was revealed from huh28 cell lysates following exposure to MC‐LR at 5, 50 or 500 nmol/L (Figure 1A). In addition, the intracellular presence of MC‐LR was also detected in huh28 cells treated with MC‐LR at 5 and 500 nmol/L for 24 hours (Figure 1B). The survival ability of huh28 cells was assessed by the flow cytometric analysis and 5‐ethynyl‐2′‐deoxyuridine (EdU) incorporation. The rates of apoptosis cells were significantly decreased after MC‐LR exposure at 0.5, 5, 50 or 500 nmol/L (Figure 1C). The EdU incorporation assay indicated huh28 cell proliferation was significantly increased following exposure to MC‐LR at 50 or 500 nmol/L for 24 hours (Figure 1D). In the contrast, no change in the migration of huh28 cells has been observed (Figure 1E). The similar phenomenon was detected in RBE cells. MC‐LR was detected in RBE cells via Western blotting (Figure S6A) and immunofluorescent staining (Figure S6B) following exposure to MC‐LR for 24 hours. In addition, MC‐LR was confirmed to promote the cell proliferation (Figure S6C) and inhibit cell apoptosis (Figure S6D) in RBE cells. Similarly, there was no change of the migration of RBE cells after treatment with MC‐LR (Figure S6E).

### 3.5 Differentially expressed mRNAs in huh28 cells treated with MC‐LR

**FIGURE 2:**
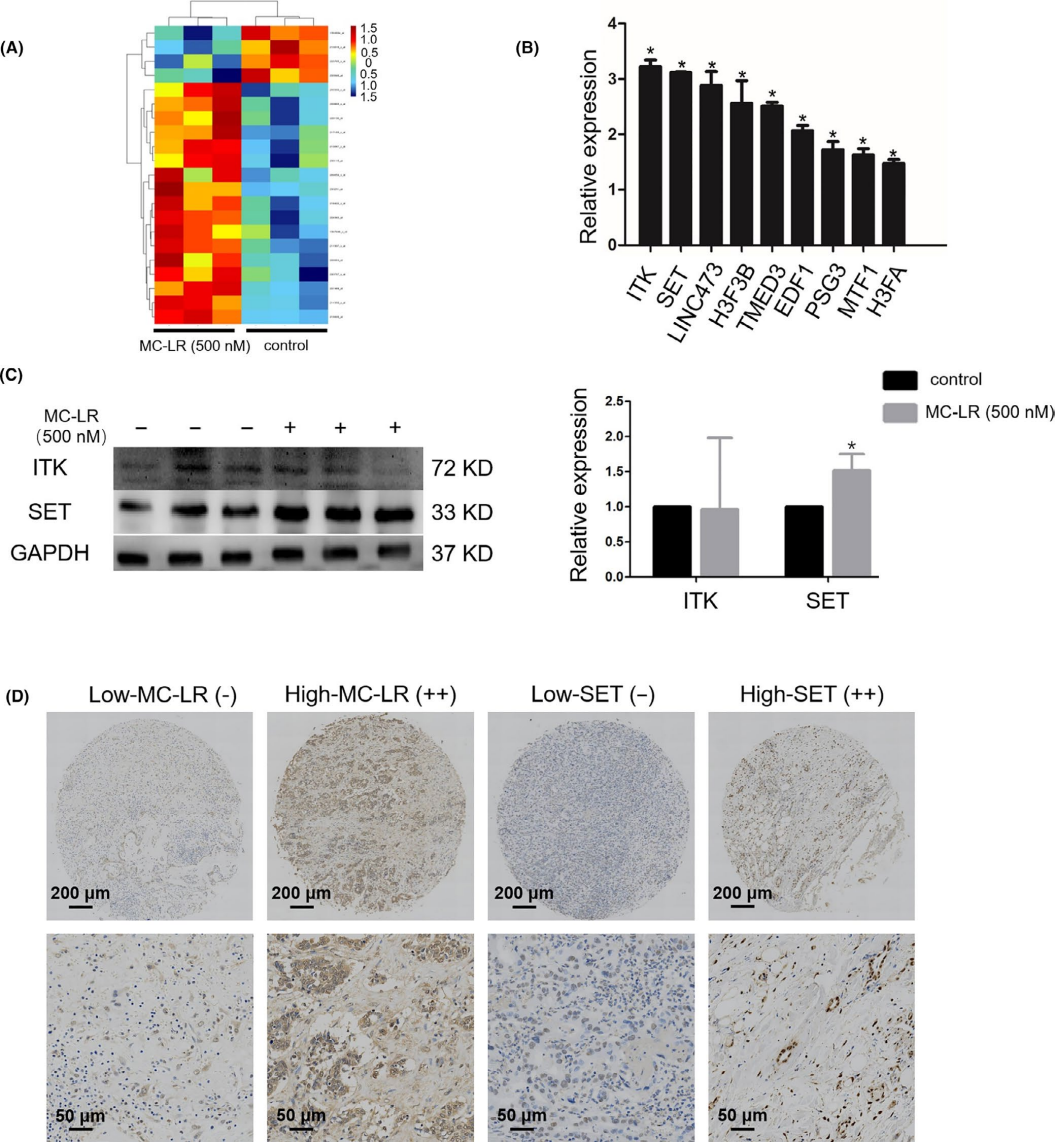
Expression of mRNAs in huh28 cells is regulated by microcystin‐leucine‐arginine (MC‐LR). A, Differentially expressed mRNAs in huh28 cells treated with MC‐LR for 24 h are shown in heat map. B, Altered expression of mRNAs (ITK, H3F3A/// H3F3B, RBM26, SET, PTCHD3P1, LINC00473, GLIPR1, ATG2B, TMED3, EDF1, ADAD1, MT1F, SPATA31A, RAD51D, PSG3, MSX2, KBTBD4, LOC283075) was confirmed by quantitative real‐time polymerase chain reaction (Q‐PCR). C, Protein levels of IL2‐inducible T‐cell kinase (ITK) and SET (I2PP2A) were measured by Western blotting. All the expression levels were quantified by densitometry and normalized to the expression of GAPDH. Data are shown as mean ± SD. Three repeats at least were performed in all experiments for three times. D, The expression levels of SET and content levels of MC‐LR were determined by immunohistochemical analyses in the tumour tissues. *P < .05 vs control

The mRNA expression profiling displayed 15 specific mRNAs with substantial changes in huh28 cells treated with MC‐LR at 500 nmol/L for 24 hours compared to those treated with vehicle (Table S2). The raw data have been deposited into Geo repository (GSE140687) (Figure 2A). We verified the expression levels of all the 15 mRNAs by Q‐PCR and found that 9 mRNAs were confirmed to be differently changed (Figure 2B). Among the significantly changed mRNAs validated, IL2‐inducible T‐cell kinase (ITK) and SET (I2PP2A) were chosen for protein level detection. The results indicated that SET protein level was increased in the huh28 cells cultured by MC‐LR, while MC‐LR show no effect to ITK protein level (Figure 2C). Besides of it, the expression of SET in RBE cells was also up‐regulated following treatment with MC‐LR (Figure S7A). We also demonstrated the expression level of SET in the tumour tissues content of high‐level MC‐LR was increased compared to that in the tissues content of low‐level MC‐LR via immunohistochemical analyses (Table S3) (Figure 2D). As a result, SET was chosen for further investigation.

### 3.6 Effect of SET protein on ICC cell proliferation induced by MC‐LR

**FIGURE 3:**
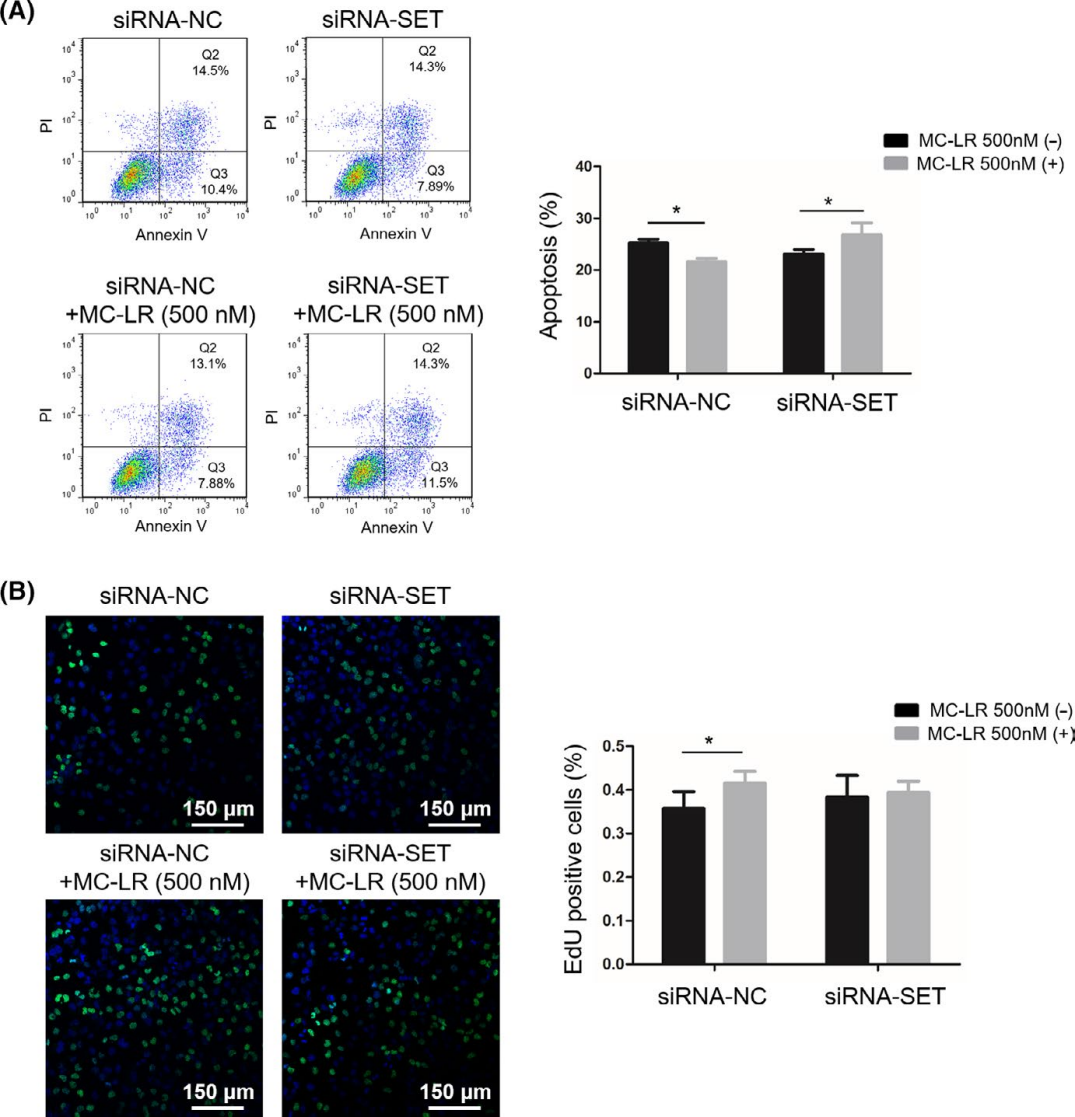
Down‐regulation of SET in huh28 cells induces cell apoptosis and reduction in cell proliferation. Huh28 cells transfected with siRNA‐NC or siRNA‐SET were treated with microcystin‐leucine‐arginine at 500 nmol/L or vehicle for 24 h. A, Cell apoptosis was analysed by flow cytometry (lower panel). B, Proliferation rates of cells transfected with siRNA were assessed by the 5‐ethynyl‐2′‐deoxyuridine (EdU) incorporation assay. Three repeats at least were performed in all experiments for three times. Data are shown as means ± SD. *P < .05 vs corresponding placebo group

To determine the effect of SET on ICC cell proliferation, ICC cells were transiently transfected with siRNA‐NC and siRNA‐SET followed by treatment with or without MC‐LR (500 nmol/L) for 24 hours. The SET protein expression was obviously decreased after the transfection with siRNA‐SET. We next explored whether down‐regulation of SET in huh28 cells influenced cell survival. Down‐regulation of SET decreased the rate of apoptosis cells induced by MC‐LR in huh28 cells (Figure 3A) and RBE cells (Figure S7B), suggesting attenuated cell apoptosis. Additionally, inhibition of cell proliferation induced by MC‐LR was confirmed by the EdU incorporation assay in huh28 cells (Figure 3B) and RBE cells (Figure S7C). There was no significantly difference of huh28 cell apoptosis and proliferation between the cells transfected with siRNA‐NC or siRNA‐SET. However, the apoptosis rate has been down‐regulated after transfection of siRNA‐SET in RBE cells compared to the cells transfected with siRNA‐NC.

### 3.7 Mechanism related analyses in the procedure

**FIGURE 4:**
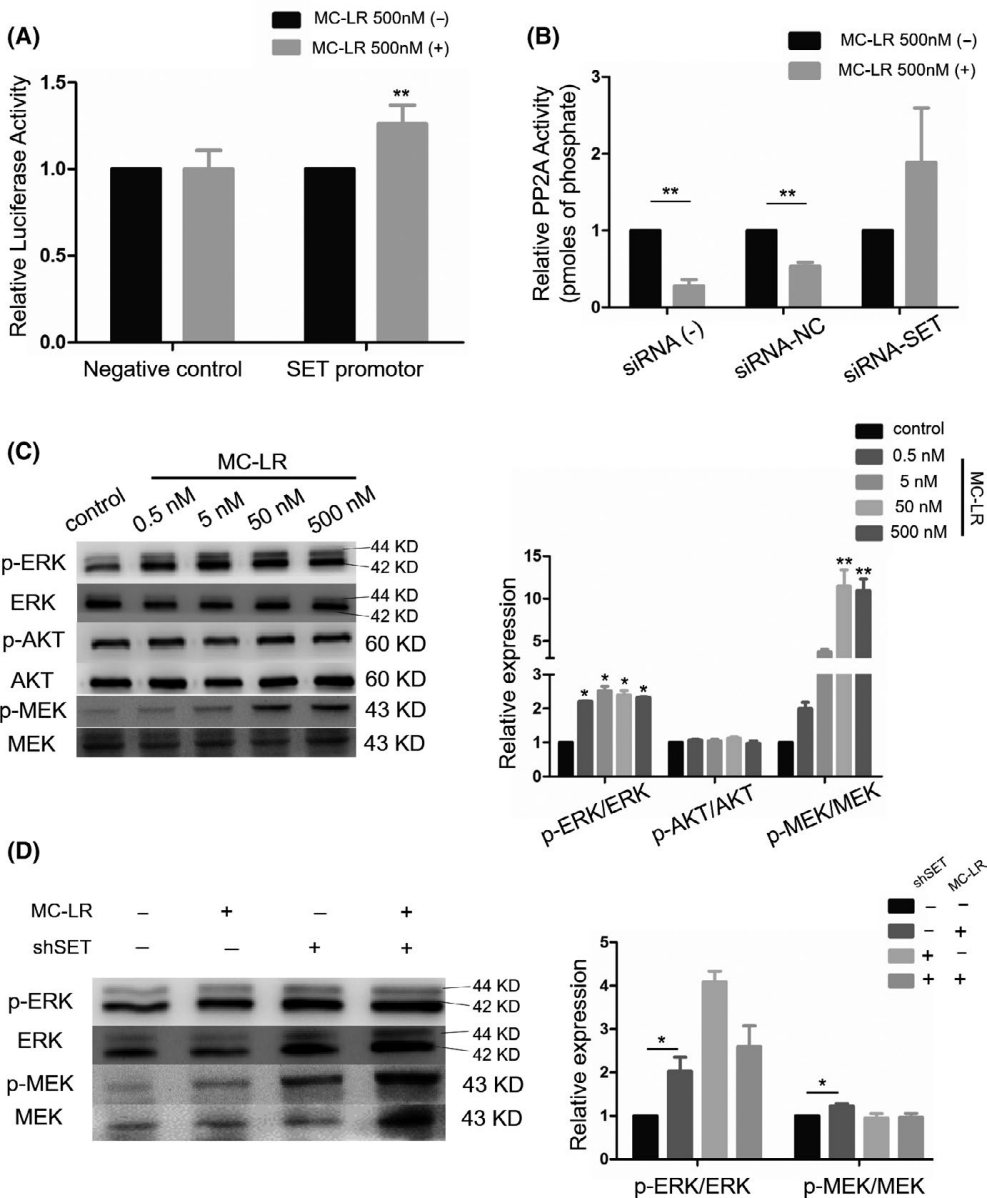
Microcystin‐leucine‐arginine (MC‐LR) inhibited the PP2A activity and activated ERK/MEK signalling pathway via regulating SET protein. A, Luciferase reporter assays were performed in 293T cells, transfected with negative control or SET‐promotor, following treatment with MC‐LR at 500 nmol/L or vehicle for 24 h. Firefly luciferase activity was normalized by the Renilla luciferase activity. **P < .01 vs placebo group. B, PP2A activity assays were determined in huh28 cells, transfected with control, siRNA‐NC and siRNA‐SET, following exposure to MC‐LR at 500 nmol/L or vehicle for 24 h. **P < .01 vs corresponding placebo group. C, Expression levels of p‐MEK1/2, MEK1/2, p‐ERK1/2, ERK1/2, p‐AKT and AKT were defined via Western blotting huh28 cells treated with MC‐LR at various concentrations. D, Expression levels of p‐ERK1/2, ERK1/2, p‐MEK1/2 and MEK1/2 in huh28 cells transfected with siRNA‐NC and siRNA‐SET, following exposure to MC‐LR at 500 nmol/L or vehicle for 24 h, were defined by Western blotting. *P < .05 vs corresponding placebo group. All the expression levels were quantified by densitometry and normalized to the expression of GAPDH. Data are shown as mean ± SD. Three repeats at least were performed in all experiments for three times

We cloned promoter sequences of SET into the pGL3 vector to demonstrate the interaction between MC‐LR and SET. The results confirmed that exposure to MC‐LR significantly increased the luciferase activity of the vector with the promoter sequences but not that of negative control, indicating MC‐LR could promote the transcriptional process of SET protein (Figure 4A). Since SET protein and MC‐LR were identified as the inhibitors of PP2A, the changes in PP2A activity of huh28 cells were determined. The results showed that the PP2A activity was inhibited after the treatment with MC‐LR (500 nmol/L) in huh28 cells transfected with siRNA‐NC. No change of PP2A activity was detected in huh28 cells transfected with siRNA‐SET after exposure to MC‐LR (Figure 4B). The similarly results were also detected in RBE cells (Figure S8A).

To determine the role of downstream signalling pathway in ICC cell proliferation induced by MC‐LR, we measured phosphorylation level of ERK1/2, MEK1/2 and AKT. The protein level of p‐ERK1/2 and p‐MEK1/2 was increased in huh28 cells treated with MC‐LR, while no change of AKT phosphorylation was observed (Figure 4C). In the meanwhile, the activation of MEK/ERK signalling path was also observed in RBE cells (Figure S8B). Moreover, after transfection with siRNA‐SET, the increased expression levels of p‐MEK1/2 and p‐ERK1/2 induced by MC‐LR were suppressed in huh28 cells (Figure 4D) and RBE cells (Figure S8C).

## 4 DISCUSSION

The World Health Organization established a guideline of 1 μg/L of MCs in drinking water. MCs pollution is a common phenomenon in humid and tropical areas in China.^24^ Besides, MCs were also detected in eutrophic lakes in California.^25^ MC‐LR has been confirmed to induce hepatocellular carcinoma using both mouse and human models.^10^, ^26^ However, little work has focused on the correlation between MC‐LR and ICC.

In our data, mass‐forming type rate of ICC in low‐MC‐LR group was significantly different compared with high‐MC‐LR group. Aishima et al reported that chronic hepatitis or cirrhosis could induce mass‐forming type ICC.^27^ In our previous study (not published), MC‐LR has been confirmed to be associated with liver fibrosis. It is also reported that, after oral chronic exposure, MC‐LR‐treated mice exhibited abnormal bile duct hyperplasia and thickened bile duct morphology, which was responsible for the cancer incidence, indicating that MC‐LR can make an influence on growth patterns of ICC.^28^ In addition, the female rate in low‐MC‐LR group was obviously decreased. It is reported that serological and pathological changes associated with liver disease in female mice were worse than male mice at the same concentration.^29^ However, Mrdjen et al could not detect the content level of MC‐LR in liver tissue. Combining our results, we considered that female mammal could accumulate more MC‐LR in liver, resulting in the high content level of MC‐LR in female patients.

Our data identified high‐MC‐LR was a prognostic indicator for ICC patients after hepatectomy. The CT values in equilibrium phase in tumour were confirmed to distinguish ICCs with different content levels of MC‐LR effectively. Chen et al^30^ have proved MC‐LR could increase vascular permeability through IL‐8/CXCR2 signalling in vivo and vitro, which might be associated with the higher CT values in tumour with high content of MC‐LR and deserved for further study.

Based on the aforementioned results, ICC cells were exposed to MC‐LR at various concentrations for further investigation. The cell proliferation of ICC cells was demonstrated by EdU staining and cell apoptosis analysis, supporting that MC‐LR can promote ICC cells survival.

Previous studies have reported that SET is related to many cellular processes because of its inhibition for PP2A and also acts as an oncogene in HNSCC^12^ and non‐small cell lung cancer.^31^ In this research, we performed a mRNA microarray and identified that SET was the most changed protein in the huh28 cells treated with MC‐LR. We discovered that restraining the expression of SET could significantly inhibit MC‐LR‐induced cell survival. These results support that SET expression regulated by MC‐LR was obviously connected with the development of ICC cells. Otherwise, overexpression of SET could inhibit cell apoptosis in RBE cells. In the previous study, overexpression of SET could inhibit the proliferation of A549 cells which was similar with the result in RBE cells.^32^ Besides of it, the transcriptional process of SET protein could be activated by MC‐LR. DNA methylation has been confirmed to be involved in the transcriptional process of various proteins.^33^ It is believed that DNA methylation might also act in the up‐regulation of SET induced by MC‐LR.

MC‐LR and SET were both believed to be the PP2A inhibitor through direct binding to the catalytic domain of PP2A in previous studies.^34^, ^35^ The MEK/ERK pathway inhibitor resistance was conferred with PP2A inhibition.^36^ However, our results indicated that the PP2A‐inhibitor activity of MC‐LR was suppressed significantly after the down‐regulation of SET in ICC cells, indicating that SET was involved in the suppression progress of PP2A induced by MC‐LR. Besides of it, the downstream MEK/ERK signalling pathway was also depressed in the ICC cells transfected with siRNA‐SET ignoring the existence of MC‐LR. Overall, these results proved that the PP2A‐inhibitor activity of MC‐LR in ICC cells was consistent with the overexpression of SET.

Our study has several limitations. First, we measured MC‐LR by self‐review using immunohistochemistry in tissue microarray blocks, which increases the possibility of information bias. Second, the connection between MC‐LR and the prognosis of ICC was analysed in a small number of individuals using retrospective data, which limited its statistical power. Last, the precise mechanisms of MC‐LR‐induced overexpression of SET had not yet discovered. Therefore, multicentre, suitably designed and prospective studies with larger sample size in several centres should be conducted to analyse the relationship between MC‐LR and ICC.

We confirmed that MC‐LR was able to inhibit the activity of PP2A and activate MEK‐ERK signalling pathway via up‐regulating the expression of SET, resulting in the ICC cell proliferation and poor prognosis. Our data indicated a potential toxin of MC‐LR as a promoter of ICC growth.

## CONFLICT OF INTEREST

Shen Gu, Wei He, Minghao Yan, Jian He, Qun Zhou, Xiaopeng Yan, Xiao Fu, Jun Chen, Xiaodong Han and Yudong Qiu declare that they have no conflict of interest.

## AUTHOR CONTRIBUTIONS

Shen Gu and Wei He were responsible for the experiments, data analysis and writing of article. Minghao Yan contributed to the experiments of study; Qun Zhou and Jian He were responsible for the collection and analysis of imaging data. Xiaopeng Yan and Xiao Fu contributed to the collection and analysis of clinical data. Jun Chen, Xiaodong Han and Yudong Qiu were responsible for the concept and design.

## Supporting information

Fig S1

Fig S2

Fig S3

Fig S4

Fig S5

Fig S6

Fig S7

Fig S8

Table S1‐S4

## Data Availability

The data that support the findings of this study are available from the corresponding author upon reasonable request.
