# Supplementary figures and images for: Higher content of microcystin‐leucine‐arginine promotes the survival of intrahepatic cholangiocarcinoma cells via regulating SET resulting in the poorer prognosis of patients
Source: Cell Prolif. 2020 Nov 25;54(2):e12961. doi: 10.1111/cpr.12961 (PMC7848955; doi:10.1111/cpr.12961)

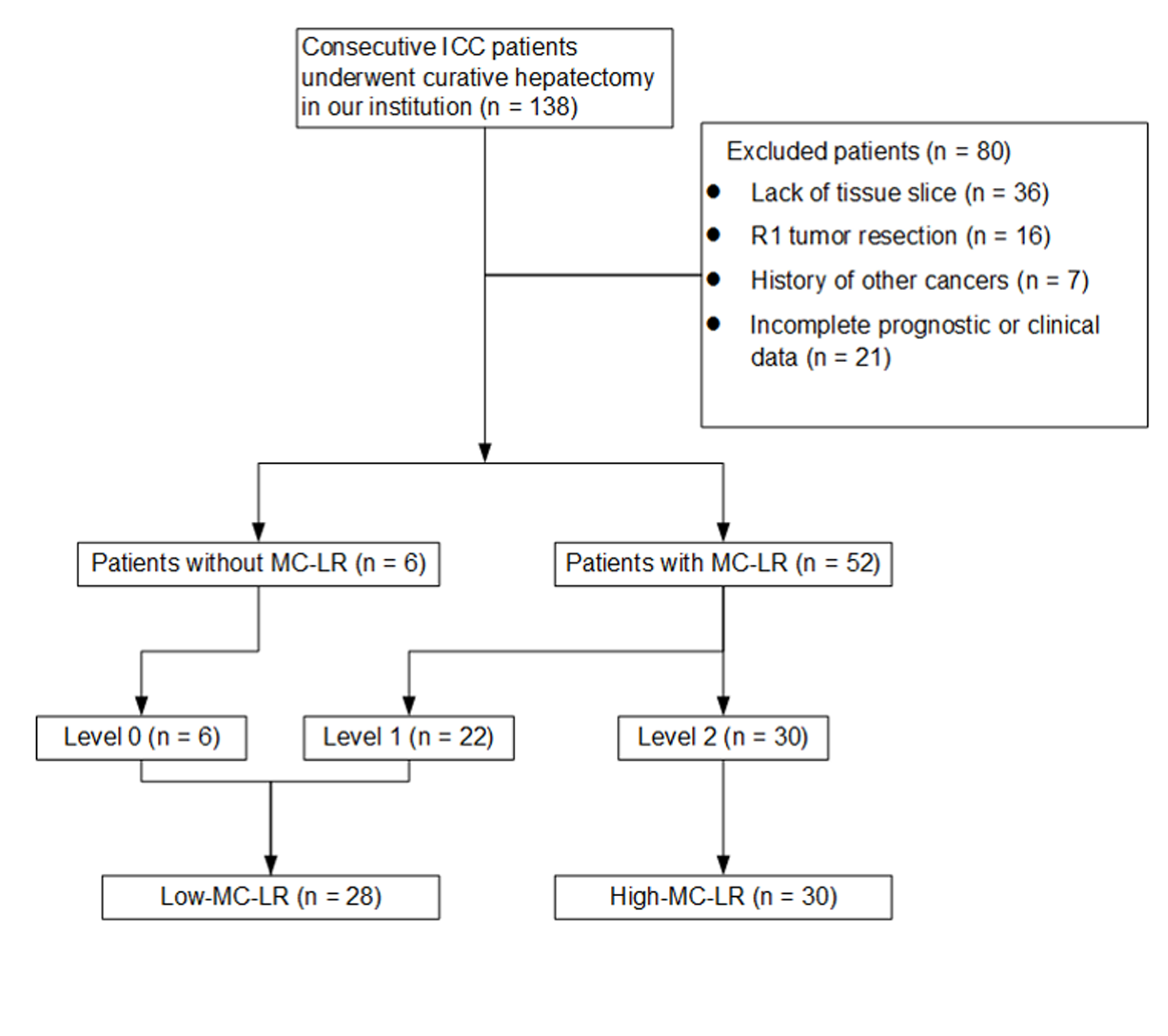

Supplement: Supplementary file 1 — Fig S1 [file CPR-54-e12961-s001.tif]

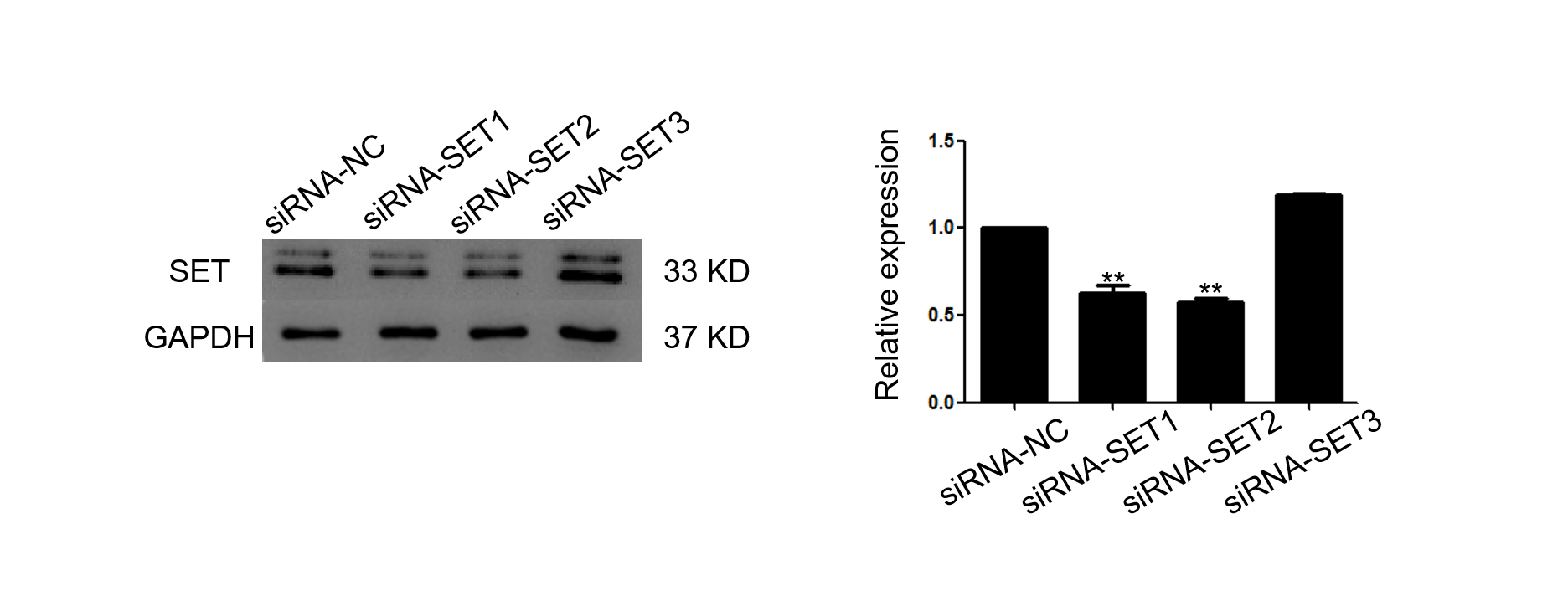

Supplement: Supplementary file 2 — Fig S2 [file CPR-54-e12961-s002.tif]

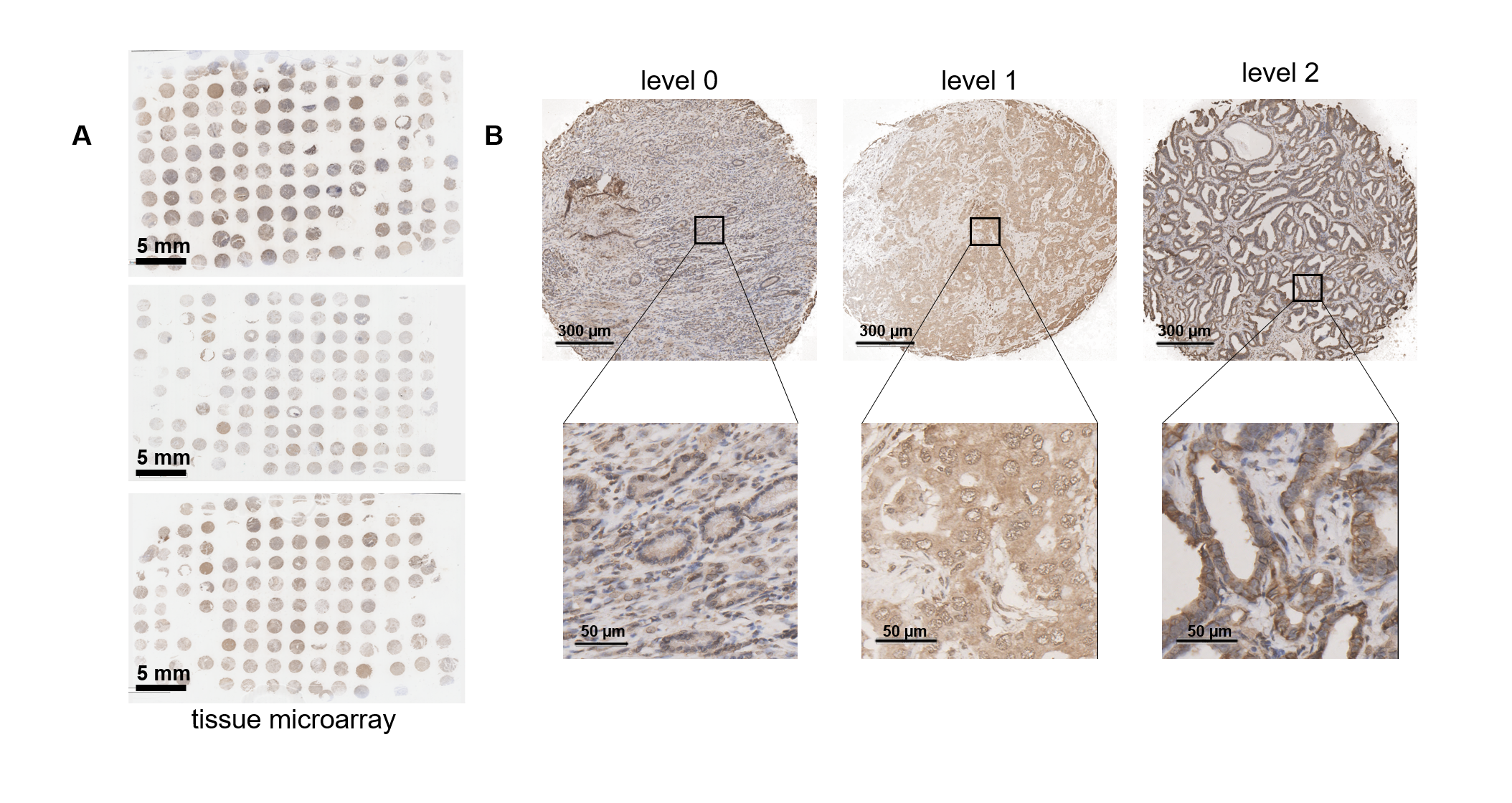

Supplement: Supplementary file 3 — Fig S3 [file CPR-54-e12961-s003.tif]

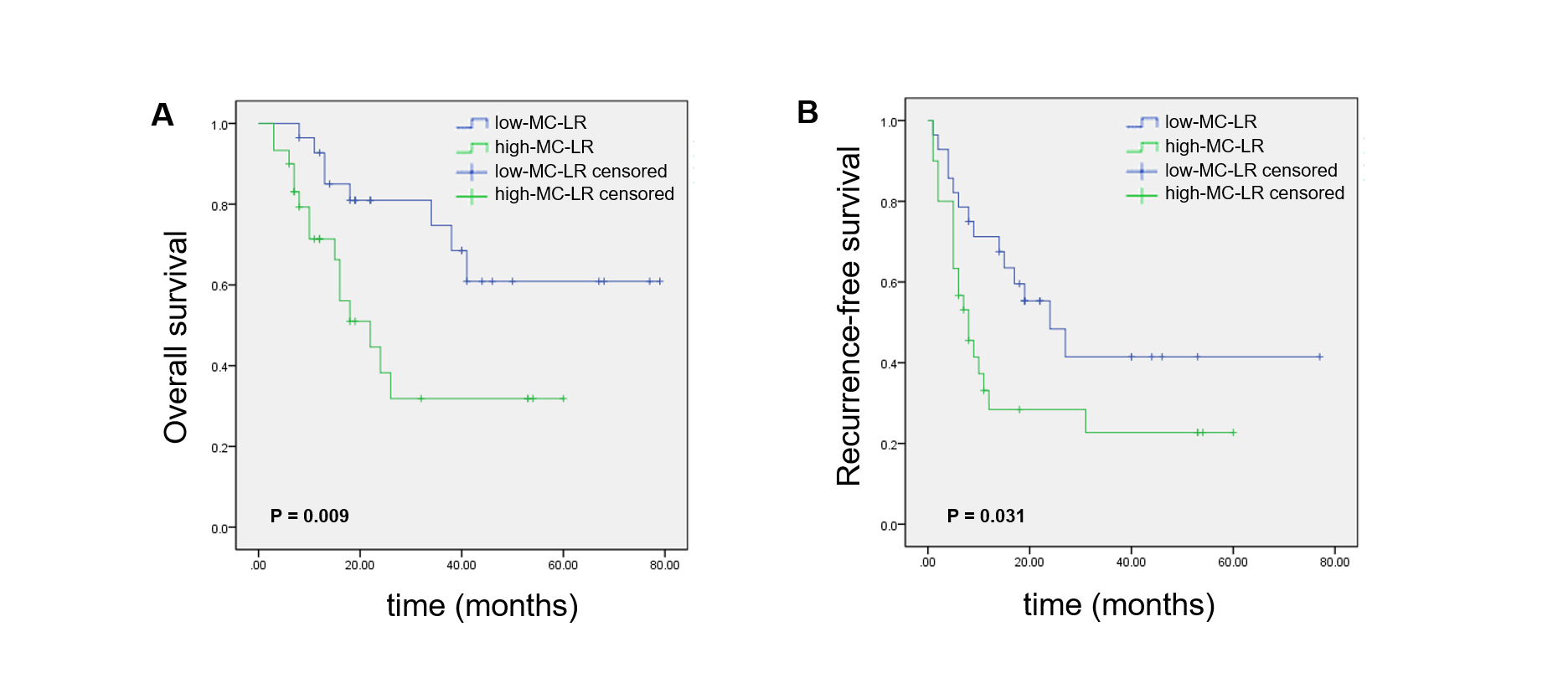

Supplement: Supplementary file 4 — Fig S4 [file CPR-54-e12961-s004.tif]

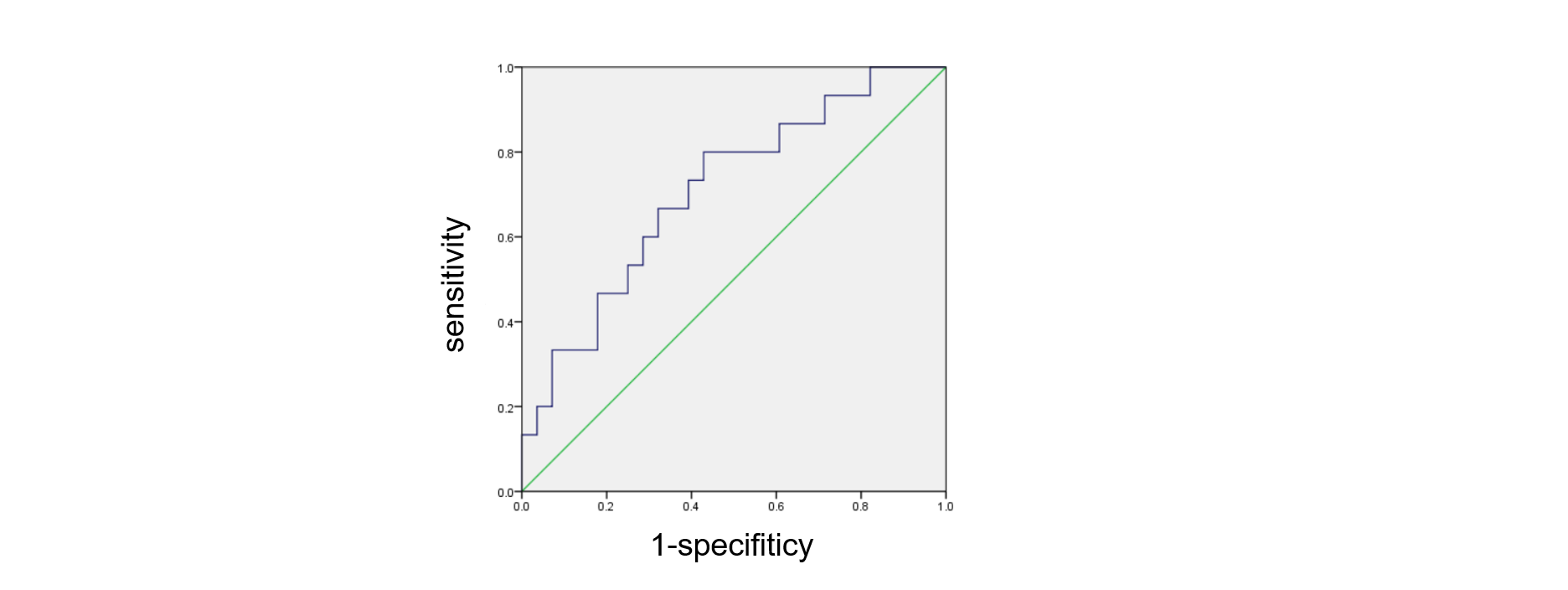

Supplement: Supplementary file 5 — Fig S5 [file CPR-54-e12961-s005.tif]

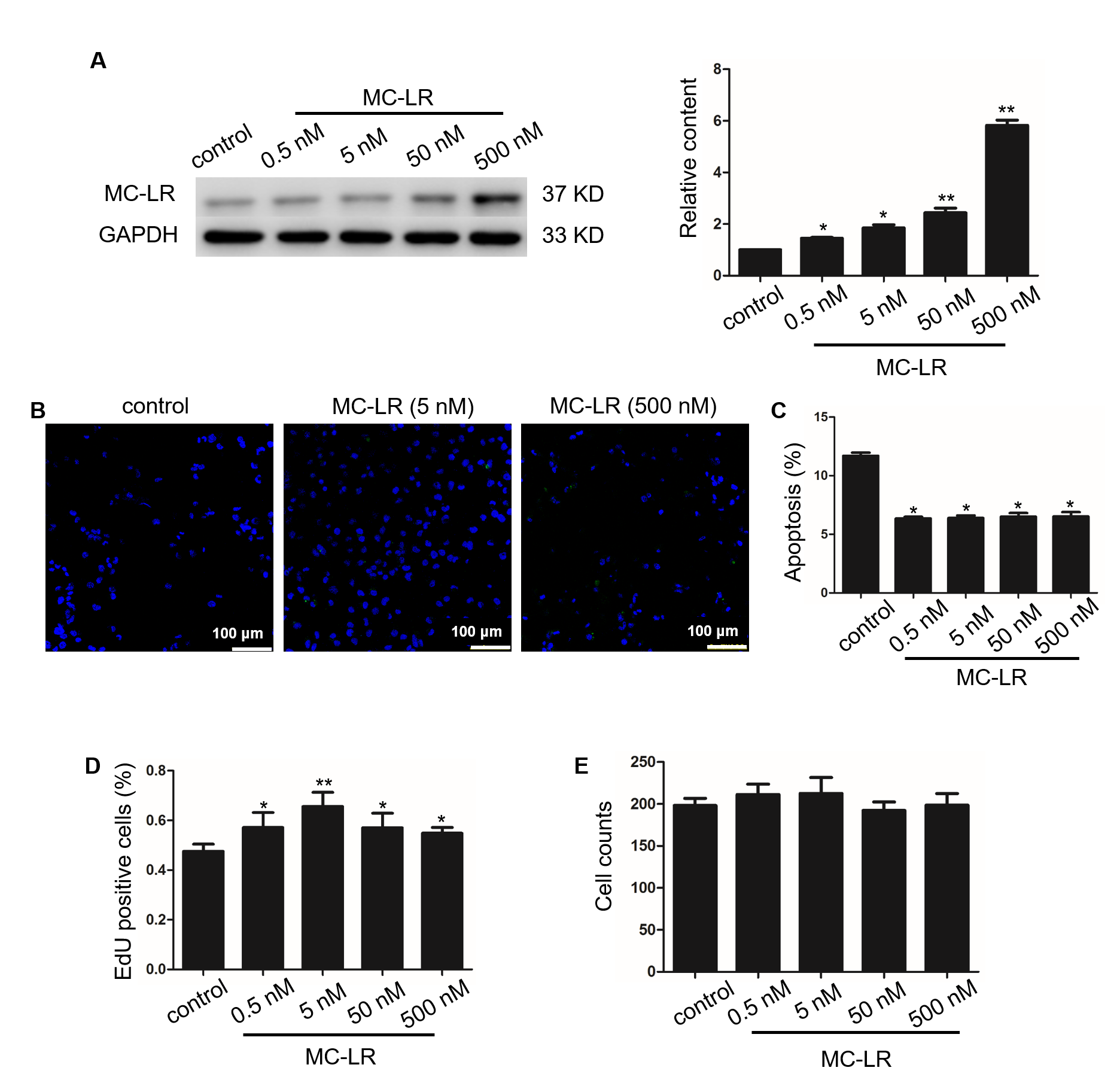

Supplement: Supplementary file 6 — Fig S6 [file CPR-54-e12961-s006.tif]

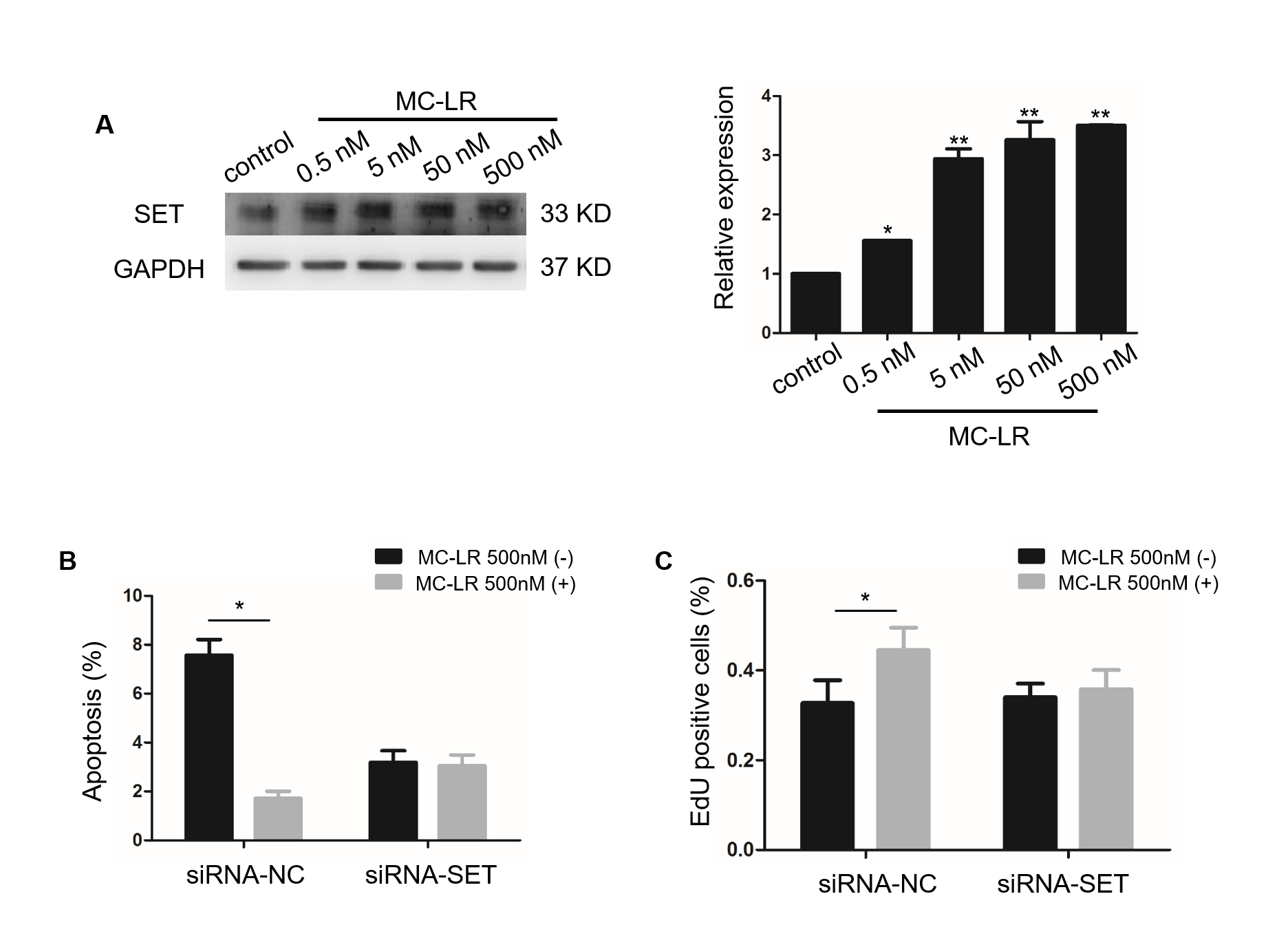

Supplement: Supplementary file 7 — Fig S7 [file CPR-54-e12961-s007.tif]

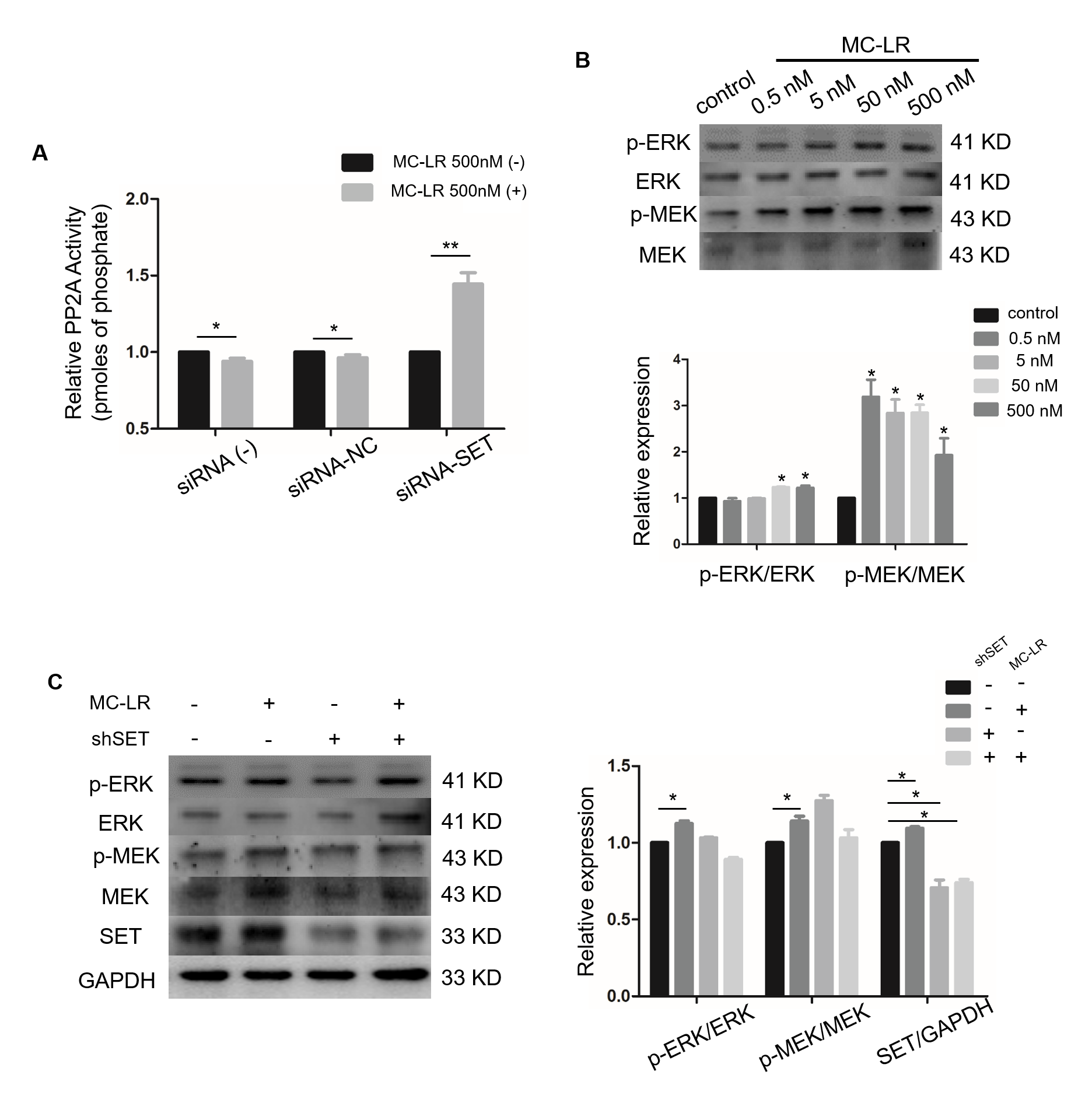

Supplement: Supplementary file 8 — Fig S8 [file CPR-54-e12961-s008.tif]
