# Supplementary material for: Higher content of microcystin‐leucine‐arginine promotes the survival of intrahepatic cholangiocarcinoma cells via regulating SET resulting in the poorer prognosis of patients
Source: Cell Prolif. 2020 Nov 25;54(2):e12961. doi: 10.1111/cpr.12961 (PMC7848955; doi:10.1111/cpr.12961)
Supplement: Supplementary file 9 — Table S1‐S4 [file CPR-54-e12961-s009.docx]

**Supplementary Tables and Figure legends**

**Table S1: Comparative analysis of characteristics among the low-MC-LR and high-MC-LR groups.**

| **Variable** | **Low-MC-LR**  **(n = 28)** | **High-MC-LR**  **(n = 30)** | **P-value** |
| --- | --- | --- | --- |
| Age (years) ^a^ | 57.5 (43-79) | 60 (34-81) | 0.319 |
| Gender |  |  |  |
| Male | 19 (68) | 12 (40) | 0.031* |
| Female | 9 (32) | 18 (60) |  |
| Background liver |  |  |  |
| Non-cirrhosis | 18 (64) | 20 (67) | 0.534 |
| Cirrhosis | 10 (36) | 10 (33) |  |
| Gross Classification |  |  |  |
| MF | 24 (86) | 19 (63) | 0.049* |
| Non-MF | 4 (14) | 11 (37) |  |
| Tumor number |  |  |  |
| Single | 23 (82) | 23 (77) | 0.426 |
| Multi | 5 (18) | 7 (23) |  |
| Longest diameter (cm) | 5.1 (0.5-12.0) | 5.1 (1.5-10.5) | 0.994 |
| Tumor differentiation |  |  |  |
| Well | 18 (64) | 17 (56) | 0.373 |
| Moderate/Poor | 10 (36) | 13 (44) |  |
| Tumor staging |  |  |  |
| I＆II | 21 (75) | 22 (37) | 0.562 |
| Ⅲ＆Ⅳ | 7 (25) | 8 (61) |  |
| Microvascular invasion |  |  |  |
| Non-MVI | 21 (75) | 18 (60) | 0.175 |
| MVI | 7 (25) | 12 (40) |  |
| Tumor stroma rate (%) ^a^ | 30 (10-70) | 30 (20-70) | 0.315 |
| ALT (U/L) ^a^ | 24.3 (8.5-342.8) | 23.6 (6.9-381.1) | 0.858 |
| AST (U/L) ^a^ | 25.55 (11.70-228.30) | 26.05 (12.60-191.80) | 0.950 |
| AKP (U/L) ^a^ | 115.55 (14.50-836.60) | 90.90 (39.80-732.40) | 0.122 |
| GGT (U/L) ^a^ | 68.50 (11.90-1004.30) | 75.55 (14.00-946.60) | 0.518 |
| LDH (U/L) ^a^ | 173.0 (123.0-259.0) | 160.5 (104.0-266.0) | 0.375 |
| TB (µmol/L) ^a^ | 11.7 (6.6-323.9) | 13.0 (6.4-104.6) | 0.669 |
| DB (µmol/L) ^a^ | 3.1 (2.0-231.3) | 3.4 (1.7-84.1) | 0.544 |
| Albumin (g/L) ^a^ | 40.10 (32.20-49.80) | 40.75 (35.30-49.40) | 0.876 |
| AFP (ng/ml) ^a^ | 2.15 (0.50-56.70) | 2.55 (0.10-3250.00) | 0.246 |
| CEA (ng/ml) ^a^ | 1.55 (0.10-88.30) | 2.25 (0.20-395.10) | 0.414 |
| CA19-9 (U/ml) ^a^ | 35.82 (0.60-18280.00) | 162.70 (0.60-25209.00) | 0.091 |

Parenthesis indicates percentage unless indicated. ^a^ median (range) * P < 0.05 for post hoc test comparison with low-MC-LR.

MC-LR, microcystins-leucine-arginine; MF, Mass-forming type; MVI, microvascular invasion; ALT, alanine aminotransferase; AST, Aspartate aminotransferase; AKP, alkaline phosphatase; GGT, gamma glutamyl transpeptidase; LDH, lactate dehydrogenase; TB, total bilirubin; DB, direct bilirubin; AFP, alpha-fetoprotein; CEA, carcinoembryonic antigen; CA19-9, carbohydrate antigen 19-9.

**Table S2: The entire list of differentially expressed mRNAs in huh28 cells treated with microcystins-leucine-arginine (MC-LR) for 24 h.**

| **Assay** | **Fold-change(MC-LR/control)** | **P-value** |
| --- | --- | --- |
| ITK | 3.404978652 | 0.001650375 |
| H3F3A /// H3F3B | 2.853622656 | 0.012606791 |
| RBM26 | 2.578344618 | 0.011494098 |
| SET | 2.524326758 | 0.024876565 |
| PTCHD3P1 | 2.367531726 | 0.037258483 |
| LINC00473 | 2.243284065 | 0.032930344 |
| GLIPR1 | 2.205430362 | 0.030335977 |
| ATG2B | 2.204364527 | 0.019768236 |
| TMED3 | 2.193917325 | 0.040614658 |
| EDF1 | 2.143649841 | 0.042851639 |
| ADAD1 | 2.099466548 | 0.001844775 |
| MT1F | 2.084531502 | 0.017713969 |
| SPATA31A | 2.075021686 | 0.00475125 |
| RAD51D | 2.031934471 | 0.013920972 |
| PSG3 | 2.007664131 | 0.016177737 |
| MSX2 | 0.452415272 | 0.016212234 |
| KBTBD4 | 0.45006246 | 0.049004013 |
| LOC283075 | 0.151512534 | 0.034153732 |

**Table S3: Chi-square analysis of association among the content level of MC-LR and SET.**

| **SET** | **MC-LR** | | |  |
| --- | --- | --- | --- | --- |
|  | **Low-MC-LR** | **High-MC-LR** | **Total** | **P-value** |
| **Low-SET** | 40 | 4 | 44 |  |
| **High-SET** | 8 | 11 | 19 |  |
| **Total** | 48 | 15 | 63 | < 0.001* |

MC-LR, microcystins-leucine-arginine.

**Table S4. The list of primers used in study.**

| **Name** | **Sequences** |
| --- | --- |
| SET qPCR primer | Forward: GCCCAAAGTCCAATTTGAGGCAGT |
|  | Reverse: ATGGTCTGGTTCTTGGACTTCCCT |
| ITK qPCR primer | Forward: TGAACAACTTTATCCTCCTGGAAGA |
|  | Reverse: GGTTAACACAAAGAAGCGGACTTTA |
| LINC00473 qPCR primer | Forward: TCATTTCCCTACCTGCTCCT |
|  | Reverse: CAGTGTCTGCACATCGCTAAT |
| H3F3A qPCR primer | Forward: CCTTGATAGAATCACTCAGTT |
|  | Reverse: ACCCCCCTTCTCCTTCGG |
| H3F3B qPCR primer | Forward: CACGGCCTTTGTGTCGGG |
|  | Reverse: TTTATAGGCACGCTTCACGC |
| GLIPR1 qPCR primer | Forward: CCGCCATCACAAACTGGTAT |
|  | Reverse: TCTGCCCAAACAACCTGAGT |
| ATG2B qPCR primer | Forward: AGTACTAGTAAGGAGAGTGGAACCAGGAG |
|  | Reverse: AGTAAGCTTCCTTTGCCAAAAACTTTCA |
| TMED3 qPCR primer | Forward: CCTCCCATTCTCCCAGACT |
|  | Reverse: CAATCGTCTCGCCAACAGAC |
| EDF1 qPCR primer | Forward: ATCTTAGCGGCACAGAGACGAGG |
|  | Reverse: GAACCGGCGGAACGAGATCAGC |
| ADAD1 qPCR primer | Forward: ACGGTTAGTGATTGGACGAAGC |
|  | Reverse: CAAAAGAGGCCTCGCGTC |
| MT1F qPCR primer | Forward: GACTGATGCCAGGACAACCT |
|  | Reverse: AGGAATGTAGCAAATGGGTCA |
| RAD51D qPCR primer | Forward: TCTCTAGGAAGGGGTAGGGGA |
|  | Reverse: CACAGTCCGACCCTGAGCA |
| PSG3 qPCR primer | Forward: TCCACAGAGGAGAACACGCA |
|  | Reverse: CTTCAATCGTGACTTCGGCA |
| KBTBD4 qPCR primer | Forward: ACAGGCAGGTAGGGAAATCG |
|  | Reverse: CTCCTCTGGTGATTCCATGCTA |
| MSX2 qPCR primer | Forward: GGAGCGGCGTGGATGCAGGAA |
|  | Reverse: AAGCACAGGTCTATGGAACGG |
| GAPDH qPCR primer | Forward: GGAGCGAGATCCCTCCAAAAT |
|  | Reverse: GGCTGTTGTCATACTTCTCATGG |

**Figure legends**

**Figure S1: Patients were enrolled according to the flow diagram.**

**Figure S2: Transfection with siRNA-SET2 effectually inhibited the expression levels of protein SET in huh28 cells.** The expression levels of protein SET were determined via Western blotting in huh28 cells, transfected with siRNA-NC, siRNA-SET1, siRNA-SET2 and siRNA-SET3. All the expression levels were quantified by densitometry and normalized to the expression of GAPDH. Data are shown as mean ± SD. Three repeats at least were performed in all experiments for three times. *P < 0.05 vs. siRNA-NC group.

**Figure S3: Microcystins-leucine-arginine (MC-LR) was defined by immunohistochemical analyses in tissue microarray blocks (TMAs) of intrahepatic cholangiocarcinoma (ICC).** (A) TMAs containing tumor sections of 141 ICC patients. (B) The content levels of MC-LR in tumor were divided into three levels: level 0 (n = 6), level 1 (n = 22) and level 2 (n = 30).

**Figure S4: Long-term survival curves of high-MC-LR (n = 30) group and low-MC-LR (n = 28) were demonstrated by Kaplan-Meier analyses.** (A) Comparison of overall survival rate: high-MC-LR vs low-MC-LR (P = 0.009). (B) Comparison of recurrence-free survival rate: high-MC-LR vs low-MC-LR (P = 0.031).

**Figure S5: The receiver operating characteristic (ROC) of tMax/tMean for predicting the content level of MC-LR.**

**Figure S6: Microcystin-leucine-arginine (MC-LR) can be uptaken into BRE cells inducing cell survival of cells.** RBE cells were cultured in the presence or the absence of MC-LR at indicated concentrations for 24 h. (A) The content level of MC-LR in RBE cells was determined by Western Blotting. (B) Intracellular MC-LR was detected by immunofluorescent confocal microscopy (200×). (C) Cell apoptosis analysis in RBE cells was conducted through flow cytometry (lower panel). (D) Measurement of cell viability was carried out with the 5-ethynyl-2′-deoxyuridine (EdU) incorporation assay. (E) The migration of the RBE cells treated with MC-LR was measured through crystal violet’s staining. MC-LR was quantified by densitometry and normalized to the expression of GAPDH. Three repeats at least were performed in all experiments for three times. *P < 0.05 vs. control.

**Figure S7: Microcystin-leucine-arginine (MC-LR) increases the expression level of SET in RBE cells and downregulation of SET resists the change induced by MC-LR.** (A) The expression level of SET in RBE cells treated with MC-LR was determined by Western blotting. SET was quantified by densitometry and normalized to the expression of GAPDH. (B) RBE cells transfected with siRNA-NC or siRNA-SET were treated with microcystin-leucine-arginine at 500 nM or vehicle for 24 h. Cell apoptosis was analyzed by flow cytometry (lower panel). (C) Proliferation rates of cells transfected with siRNA were assessed by the 5-ethynyl-2′-deoxyuridine (EdU) incorporation assay. Three repeats at least were performed in all experiments for three times. Data are shown as means ± SD. *P < 0.05, **P vs. < 0.01 corresponding placebo group.

**Figure S8: Microcystin-leucine-arginine (MC-LR) inhibited the PP2A activity and activated ERK/MEK signaling pathway in RBE cells.** (A) PP2A activity assays were determined in RBE cells, transfected with control, siRNA-NC and siRNA-SET, following exposure to MC-LR at 500 nM or vehicle for 24 h. **P < 0.01 vs. corresponding placebo group (B) Expression levels of p-MEK1/2, MEK1/2, p-ERK1/2, ERK1/2 were defined via Western blotting in RBE cells treated with MC-LR at various concentrations. (C) Expression levels of p-ERK1/2, ERK1/2, p-MEK1/2, MEK1/2 and SET in RBE cells transfected with siRNA-NC and siRNA-SET, following exposure to MC-LR at 500 nM or vehicle for 24 h, were defined by Western blotting. *P < 0.05 vs. corresponding placebo group. All the expression levels were quantified by densitometry and normalized to the expression of GAPDH. Data are shown as mean ± SD. Three repeats at least were performed in all experiments for three times.

**Appendix S1. Supplementary Methods**

**Course for patients.** The follow-up ended on July 8th, 2017. All of the clinical events, such as adjuvant treatment, tumor recurrence, death and so on, were recorded at least once six months. None of the patients included in this study was lost to follow-up. The survival time was measured since they received the surgical resection. "OS" means overall survival and "RFS" means recurrence-free survival.

**Tissue microarray blocks (TMAs).** The tissue microarray containing 141 iCCA patient samples (randomly collected from February 2005 to March 2016) was used to test the content levels of MC-LR by immunohistochemistry. Tissue microarray blocks (TMAs) were constructed from the most representative tumor areas and consisted of three cores of tumor samples with diameters of 0.1 cm. All samples were from resected liver specimens. Clinical information was retrieved from electric medical records. Clinical staging was estimated according to the American Joint Committee on Cancer, 8th grading system.

**Imaging techniques.** All patients underwent CT examination in a supine position with a multidetector spiral CT scanner (Lightspeed, VCT, or Discovery HD750, GE Healthcare, Milwaukee, WI, USA). The scan ranged from the diaphragm to the pubic symphysis. After a plain CT scan, patients received 1.2 ml/kg body weight of contrast media (Omnipaque 350 mg I/ml, GE Healthcare) at a rate of 3.0 ml/s followed by 40 ml saline solution through the elbow vein using a power injector (Medrad Stellant, Indianola, PA, USA) at a rate of 3.0 ml/s. The arterial phase was obtained with a delay time of 35 s, portal venous phase with 70 s and equilibrium phase with 3 min. CT images were obtained with a tube voltage of 120 kVp, a tube current of 240 mA, a slice thickness of 5 mm, a slice interval of 5 mm, a reconstruction slice of 1.25 mm, a rotation time of 0.6 s, a helical pitch of 1.375, a field of view of 35–40 cm, a matrix of 512 × 512, and a standard reconstruction algorithm.

**Imaging analysis.** Two radiologists (Z.Y. and Z.Q., with 3 and 5 years’ experience in abdominal radiology, respectively), who were blinded to clinicopathological information, interpreted CT images independently. If there was inconsistency, consensus was achieved through discussion or referral to a third radiologist (H.J., with 10 years’ experience in abdominal radiology).

An oval region of interest (ROI) was placed in the solid part of the tumor showing the most remarkable enhancement as large as possible avoiding visible vessels, necrosis and calcifications in the lesion. Thereafter a round ROI as large as possible was placed within the abdominal aorta at the same level as the tumor. The ROIs of the tumor were kept identical in equilibrium phases. Mean CT attenuation of each ROI was obtained in hounsfield units. Each observer measured three times and the average value was obtained. The mean values of the two radiologists were calculated as the final results. Enhanced ratio in the equilibrium phases was the tumor CT value versus the CT value of the abdominal aorta. The max CT value of tumor in equilibrium and mean CT value of tumor in equilibrium phases were abbreviate to tMax and tMean separately.

**Immunohistochemical analyses.** The tissue sections were treated with 3% H_2_O_2_ for 10 min and blocked with 3% BSA for 20 min at 37°C, followed by incubation with mouse anti-MC-LR at 4 °C overnight. The target proteins were incubated with HRP-conjugated secondary antibodies at 37°C for 1 h, and then the specific binding was examined using diaminobenzidine (DAB) method according to the manufacturer's instructions (Zhongshan Biotechnology, Beijing, China). The immunohistochemical analysis was performed by 2 independent investigators. The expression level of proteins was defined according to the rate of positive cells in the field of vision.

**Cell culture.** Huh28 and RBE cells were cultured in high DMEM (GIBCO) with 10% FBS (GIBCO), 1% L-glutamine, and a 1% solution of penicillin and streptomycin. Cells were routinely passaged using 0.25% trypsin when they reached 80% confluence, and were diluted 1:2 at each passage.

**Flow cytometric analysis.** Cells were collected and suspended in 0.5 mL of 1× binding buffer and washed twice with ice-cold PBS. The annexin V–FITC Apoptosis Detection Kit (Vazyme) was used according to the manufacturer’s instructions for the apoptosis assay. After staining, samples were analyzed for cell apoptosis distribution with a FACScallbur flow cytometer (Becton Dickinson, San Jose, CA). Data were analyzed using Flow Jo. Experiments were performed independently at least thrice in duplicates.

**EdU incorporation.** Cells were subsequently stimulated for 24 h in the medium supplemented with various concentrations of MC-LR and DMEM. The EdU solution was added to the medium during the last 4 h of the 24 h incubation period. After a 24 h incubation, EdU incorporation was detected following the manufacturer's instructions. At least 5000 cells in 10 random-view fields were counted per experiment.

**Cell migration assays.** For the migration assay, 1×10^5^ cells were added into the upper chamber of an 8-μm pore sized polycarbonate nucleopore filter inserts in a 24-well Transwell chamber (Corning Costar, Cambridge, MA, USA). The lower chamber was filled with 500 µl complete medium containing 10% FBS. After incubation at 37̊C with 5% CO2 for 24 h, invaded cells on the lower surface of the membrane were fixed with ethanol and noninvasive cells were removed with a cotton swab. Then cells were stained with crystal violet. Migrated cells from five fields were counted under a microscope.

**Quantitative real-time polymerase chain reaction (Q-PCR).** Reverse transcription was performed using the Superscript III first-strand synthesis system (Invitrogen) on a Veriti 96-Well Fast Thermal Cycler (Applied Biosystems, Grand Island, NY). Q-PCR was conducted by amplifying 20 μl of diluted cDNA with the SYBR Green Q-PCR kit (Roche, Germany) on 7300 Real-Time PCR System (ABI, America). Total RNA was isolated from cells in three independent culture plates and analyzed in triplicate using Q-PCR. All the procedures were repeated for three times. The relative quantification values for each mRNA were calculated by the 2−ΔΔCt method using GAPDH as an internal reference, respectively.

**Selected small interfering RNA and transfection condition.** The negative control of siRNA (siRNA-NC), with random sequences, did not target any known mammalian gene. The siRNAs were chemically synthesized by Sangon Biotech (Shanghai, China). The preliminary studies of the project also showed that 100 nmol/L siRNA carried by Lipofectamine 2000 (Invitrogen, Thermo Fisher Scientific, USA) could significantly down-regulate the protein expression of the SET protein in huh28 and RBE cells at 48 h after transfection, and thus the same transfection conditions were used in this research.

**PP2A activity assay.** To immunoprecipitate PP2A, lysates containing 200 mg of protein were incubated with 4 μg of anti-PP2A, C subunit antibody and 40 μl of protein A-agarose slurry for 1 h at 4°C with constant rocking. The immunoprecipitates were washed three times in Tris-buffered saline and once with Ser/Thr assay buffer. The reaction was initiated by the addition of 60 μl of phosphopeptide substrate. Following incubation at 30°C for 10 min in a shaking incubator, the reaction mixture was centrifuged briefly, and the supernatant was transferred to a 96-well microtiter plate. The reaction was terminated by adding malachite green phosphate detection solution for 15 min at room temperature, and free phosphate was quantified by measuring the absorbance of the mixture at 650 nm using a microplate reader.

**Statistical analysis.** Continuous variables were compared using the Mann-Whitney U test with Bonferroni correction followed by a post hoc test while the chi-square test was used for the analysis of categorical data. The overall survival (OS) and recurrence-free survival (RFS) rates were calculated according to the Kaplan-Meier survival curves and compared by the log-rank test. Prognostic risk factors were analyzed via univariate and multivariate Cox proportional hazards models. Clinical characteristics were statistically significant in univariate analysis were subsequently included in a multivariate analysis. The image predictors for MC-LR were identified by univariate logistic regression analysis. Subsequently, the significant predictors (P < 0.10) were evaluated by multivariate logistic regression analysis to identify the valuable independent predictors for MC-LR. The cut-off value of predictive score was calculated by receiver operating characteristic (ROC) curve. Experimental results were expressed as mean ± standard deviation. Differences were analyzed by one-way ANOVA.
